# Supplementary material for: CD8+ cells and small viral reservoirs facilitate post-ART control of SIV replication in M3+ Mauritian cynomolgus macaques initiated on ART two weeks post-infection
Source: PLoS Pathog. 2023 Sep 25;19(9):e1011676. doi: 10.1371/journal.ppat.1011676 (PMC10553806; doi:10.1371/journal.ppat.1011676)
Supplement: Attachment — Submitted filename: Responses to Reviewers.docx [file ppat.1011676.s009.docx]

# Responses to Reviewer #1:

This manuscript by Harwood et. al describes a novel model of post treatment control (PTC) in SIV infected Mauritian cynomologous macaques (MCMs). Mechanisms underlying post treatment control have been difficult to investigate, due to its rare occurrence in both people living with HIV and in the most commonly used nonhuman primate model, SIV infection of rhesus macaques (RhMs). In MCMs that were infected with SIVmac239M and started on cART at two weeks post infection, 7 of 8 animals qualified as PTCs after cART was interrupted after 8 months. In MCMs that were infected with SIVmac239M and started cART at eight weeks post infection 2 of 6 animals were classified as PTCs upon cART release after 20 months of treatment. The authors recategorized animals from both cohorts as PTCs, blippers, or rebounders for subsequent analyses. They found that PTC status was associated with preserved CD4+ frequencies and lower frequencies of CD8+ T cells expressing exhaustion markers, but not bulk CD8 T cell frequencies, CD4+ T cell frequencies prior to cART withdrawal, frequencies of CD4+ or CD8+ T cells expressing cytokines and cytolytic markers, differential gene expression in CD8+ T cells, or cell associated viral RNA in lymph node samples on cART or post cART, or anti-SIVmac239 Env antibodies. Monoclonal antibody mediated depletion of CD8a cells in all eight of the 2 week cART group resulted in a significant increase in plasma viremia, indicating that virus suppression was mediated by CD8a cells. Interestingly, the authors showed that MCMs had significantly less total and intact vDNA at 2 weeks post infection than rhesus macaques infected with the same virus, dose, and route, which coincided with an approximate one log difference in plasma viral loads at 2wpi despite having similar viral loads at peak just a few days prior.

Given the results of this study and the limited MHC diversity in MCMs, this model provides an exciting opportunity for further investigation into the development of post treatment control. Additionally, the observation of an approximate one log reduction in plasma viral loads at 2wpi relative to SIVmac239 infected animals is another interesting potential area of research, as it suggests MCMs are able to exhibit some measure of control over acute phase SIVmac239 replication to a greater extent than RhMs. Overall, this is a well conducted study in an intriguing area of the field and the authors do a good job exploring how different aspects of this model could be further manipulated to elucidate the interplay between the virus and the immune system.

We thank this reviewer for the meticulous review of this manuscript, and the insightful comments and suggestions. We have addressed the comments raised by this reviewer in the following ways (in blue):

Major Comments:

1. One result that I found particularly interesting, but don’t feel the authors discussed enough, was the finding that there was no difference in lymph node cell associated viral RNA post cART across the PTC, blipper, and rebound groups, despite distinct plasma viral load profiles. This suggests that there is an effective immune response that is containing viral replication in the PTCs and blippers in this compartment that isn’t present in the rebounders. While the authors had lymph node homogenates for the vRNA measurements, they do not address whether assays for cellular responses could be conducted on additional LN cells from this biopsy. Understanding the lymph node resident CD8+ T cell response is key given this result, especially in light of the fact that analyses of blood CD8+ T cells only showed a difference in expression of exhaustion markers, but not much of a difference in expression of functional cytokines and cytolytic markers. I would encourage the authors to conduct these analyses on cryopreserved LN cell samples if they are available, and if they are not to add a paragraph in the discussion addressing the discordant pVL vs LN vRNA result and the possible role of tissue resident CD8+ T cells.

We thank the reviewer for this comment, and we agree it is an excellent suggestion. It is interesting that the MCMs had such disparate post-ART viral load profiles despite similar amounts of cell-associated viral RNA in the LNs, and we did not sufficiently discuss this observation in the previous version. Unfortunately, limitations on sample availability prevent us from performing additional lymph node analyses in the present study for two reasons. First, LNs from the animals that previously received therapeutic interventions were heavily sampled and assayed during the previous therapeutic intervention study ([PMC9749465](http://www.ncbi.nlm.nih.gov/pmc/articles/pmc9749465/)) that included N-803 treatment (which occurred 3 days to 4 weeks post-ART withdrawal), and this intervention would likely drastically impact functional cellular analyses during this time. This would confound analyses aimed at understanding the discordant viral load outcomes regardless of previous treatment group. Second, due to the heavy sample usage from the original therapeutic intervention study, samples from many of the animals were depleted, so LN analyses from all animals included here is not possible. We do agree this is an important point to address in future studies of PTC in MCMs, and we have raised this point and added some appropriate references in the discussion, lines 291-300.

Minor Comments:

1. Line 113: Suggest this sentence include a reference to M6, e.g. “None of these MCMs possessed the M1 or M6 MHC haplotypes associated with SIV control (Table 1).”

We have adjusted that sentence and included the reference to Mee et al 2009, which describes the M6 haplotype as protective.

2. Line 116, lines 339-346: It would be nice to include at least a sentence or two describing the therapeutic vaccination given to four of the animals, to provide a bit of context for the reader, rather than making them access the reference. Namely, timing of vaccination relative to infection, relative to cART release, and results of vaccination. While the current results did not seem to suggest that the vaccination had an impact on PTC outcome, the reader should have enough information to think about whether this treatment may have impacted the current study in any way.

We agree and have added the suggested details and discussion points to lines 117-123.

3. Line 147: Suggest adding the phrase “in all animals” after “increase in SIV viremia”.

We have added that clarifying phrase (new line number 153).

# Responses to Reviewer #2:

Harwood and colleagues carry out a detailed analysis of the phenomenon of post-treatment control (PTC) in Mauritian cynomolgus macaques (MCMs) infected with SIV. Unlike SIV-infected rhesus macaques (and HIV-infected humans), control of viremia after release of antiretroviral therapy (ART) was observed in a high frequency (7/8) of MCMs. Interestingly, none of these animals had MHC haplotypes previously associated with SIV control, although all expressed the M3 haplotype. Using an SIV-specific IPDA, the authors documented significantly lower level of the SIV reservoir in the MCMs compared with rhesus macaques. Furthermore, depletion of CD8alpha-expressing cells resulted in a significant rebound of viremia in the MCMs. Based on these observations, the authors conclude that the combination of relatively lower reservoirs and immune-mediated suppression contribute to the PTC phenotype in MCMs. These initial observations are provocative and support further study of the model. However, several additional studies will be required to better understand the mechanisms involved in PTC in this model. In addition, the extent to which results in this model may apply to HIV-infected humans remains uncertain.

We thank this reviewer for the thoughtful review of this article and suggestions of additional relevant information to include and further clarify. We have addressed the comments raised by this reviewer in the following ways (in blue):

Major Comments:

1. Page 6, lines 114-115: The authors state for the 2-week group they only included animals carrying the M3 haplotype, but do not provide any justification for this decision. Was the inclusion of only M3 animals in the 2-week cohort by chance or by design? And if by design, what was the rationale? In several instances (p. 2, line 24; p. 16, line 356) the authors qualify their results to M3+ haplotype MCMs, but they present no data (and there is no discussion) on whether this is a phenomenon specific to M3 haplotyope MCM or whether it may occur in other MCM as well. This issue should be explicitly addressed by the authors.

We appreciate this feedback to clarify our justification of including M3+ animals while excluding M1+ and M6+ animals and discuss how this may impact the interpretation of our results. This animal selection criteria was chosen to include only animals who had MHC haplotypes that were not known to be associated with spontaneous viral control. We anticipated that because the M3 haplotype has not been previously associated with elite control but the M1 and M6 haplotypes have, that the animals in this study would NOT be predisposed to become spontaneous SIV controllers. Because of this study design, we were even more surprised that these M3+ MCMs became PTCs. We have included a sentence with this justification in lines 114-117.

The reviewer appropriately points out that because ⅞ of the M3+ MCMs became PTCs, then the phenotype we observed may be attributed to a unique genetic feature of the M3 MHC haplotype. It is possible that the MHC genomics associated with spontaneous control are not the same as those that promote PTC. We have added a brief discussion of this possibility in lines 356-358 within the discussion section.

2. Although the authors present data and implicate cellular immune responses in PTC, no data are presented regarding SIV-specific neutralizing antibody responses. Such data would considerably strengthen the manuscript.

We thank the reviewer for this suggestion. We agree that it would be interesting to look at SIV-specific neutralizing antibodies in the MCM_2wk cohort, the MCM_8wk cohort, and the rhesus macaques presented to determine whether there are higher frequencies of neutralizing antibodies in the PTCs than the rebounders or the rhesus macaques, and whether neutralizing antibodies directly contribute to the mechanism of PTC. Unfortunately, matched serum samples are not available among these three cohorts of animals because chronic samples from the rhesus macaques that never received ART would not match samples from the MCM cohorts that received ART. Further, matched serum samples between the MCM_2wk and MCM_8wk cohorts after ART withdrawal are not available.

While Wu and colleagues recently observed a direct correlation between Env-specific antibody titers and time to rebound in four MCMs infected with SIVmac239 (Wu et al. Immunity 2023), we did not observe any differences in bulk anti-SIVmac239 gp120 IgG antibodies in MCMs that did and did not become PTCs (supplemental figure 3). These discordant results could be due to a number of factors, such as differences in the assays used by Wu and colleagues and the assay we used, differences in animal study designs, and differences in MCM MHC. It would be interesting to evaluate both anti-Env antibody responses and neutralizing antibody responses further in additional cohorts of MCMs, particularly where matched samples can be taken. However, SIVmac239 is highly neutralization-resistant, so we do not anticipate that antibody-mediated neutralization significantly contributed to the observed virus suppression. In a previous study by our group, MCMs infected with a live attenuated SIVmac239 did not exhibit significant anti-SIVmac239 neutralization potency either before or after CD8β depletion (Sutton et al. Journal of Virology 2019 [PMC6639280](http://www.ncbi.nlm.nih.gov/pmc/articles/pmc6639280/)). All of these animals expressed either one or two copies of the M3 haplotype, and none expressed the M1 or M6 haplotypes. In line with the observation of many groups that macaques typically do not generate antibodies that neutralize SIVmac239, this previous study suggests that M3+ MCMs also do not generate potent anti-SIVmac239 neutralizing antibodies, so we do not anticipate that the animals described in the present study would exhibit exceptional neutralizing antibody activity either. We have added a discussion of both anti-SIV Env antibody responses and the potential contribution of neutralizing antibody responses to PTC in lines 309-322 for the readers to consider.

3. Given the data suggesting a smaller intact reservoir in SIV-infected MCMs compared with rhesus macaques, efforts to analyze the size of SIV-susceptible target cells in both species would be of interest, including analysis in peripheral blood, lymph node, and gut-associated lymphoid tissue. Do the two species differ in percentages of memory, activated CD4+ T cells or CCR5+/CD4+ T cells?

We agree that determining the species-specific differences between rhesus macaques and MCMs, particularly regarding the availability of susceptible target cells, will be critical to more fully understand why MCMs appear to form smaller viral reservoirs compared to RMs. We have begun to explore some of these differences, and have included some additional data in Figure 2 to address these questions. Figure 2H-L now shows CD4+ T cell frequencies before SIV infection in MCMs (black) and rhesus macaques (purple) (H), the distribution of CD4+ T cell memory subsets (I), frequency of CCR5+ CD4+ T cells (J), and CCR5 mean fluorescence intensity (MFI) on bulk CD4+ T cells (K and L).

Interestingly MCMs had lower frequencies of CD4+ cells than rhesus macaques prior to SIV infection (H), with a bias toward effector and transitional memory CD4+ T cells (I). Although MCMs and RMs had similar frequencies of CCR5+ CD4+ cells (J), SIV-naïve MCMs had a lower abundance of CCR5 receptors (measured by MFI) on the surface of CD4+ T cells than SIV-naïve RMs (K, sample histogram is shown in L). These data provide some evidence suggesting that MCMs may have fewer target cells available to be infected than rhesus macaques during acute SIV, which could help explain the differential reservoir sizes between RMs and MCMs. We are performing more experiments to more fully understand the natural phenotypic differences between RM and MCM lymphocytes, the impact of SIV on these lymphocyte populations, and the association between different lymphocyte characteristics (such as memory phenotype and CCR5 expression) and reservoir formation for future manuscripts, but we agree that it is important to include this preliminary data that suggests that MCMs may harbor less infectable CD4+ T cell populations. We have altered the figure 2 legend (lines 1058-1070), results (lines 183-193), and discussion (lines 329-330) to include a description of these additional graphs to highlight these exciting results and address the questions raised by the reviewer.

Of note, these experiments were conducted using peripheral blood. Unfortunately, lymph node samples have been depleted for many of the animals described in the present study, and matched lymph node samples were not available for all animal cohorts. Gut-associated lymphoid tissue was also not collected from the animals in the present study. We agree that analyzing the cellular and reservoir dynamics in these compartments will be imperative to fully elucidate the mechanism of small reservoir formation in MCMs.

4. Page 9, lines 194-199: Are the differences in the percentage of animals exhibiting sustained plasma viremia significantly different between the 2-week MCM cohort and the 8-week MCM cohort?

Seven of the eight MCM_2wk animals (87.5%) became PTCs while only two of the six MCM_8wk animals (33.3%) became PTCs. Due to low animal numbers and the fact that the MCM_8wk animals could not be tracked beyond 12 weeks, we are unable to comment on whether there are statistical differences in the number of PTCs between these two cohorts. However, we have noted the percentages of each cohort that did and did not become PTCs in lines 124, 126, 219, and 221.

Minor Comments:

1. Page 6, lines 115-116: the specific therapeutic vaccination regimen should be briefly described in the text.

We agree, adding these details regarding previous interventions received by some of the animals is important to enable the readers to more critically evaluate the results shown here. We have added details regarding the therapeutic regimen to lines 117-123.

2. The issue of whether the CD8alpha-specific monoclonal antibody used for flow cytometry recognizes a distinct epitope from the MT807R1 antibody used for in vivo depletion should be explicitly noted.

We agree this is a critical distinction that we considered in designing the flow cytometry panels used here. We have explicitly noted that the DK25 clone of the anti-CD8α antibody was used in flow cytometry to avoid epitope masking by the CD8α depleting antibody in the methods section (lines 630-634) and included the appropriate references.

# Responses to Reviewer #3, Dr. Cristian Apetrei:

This is an excellent manuscript from a very well-versed group that actually pioneered the Mauritian cynomolgus macaque model for HIV infection. Here they report that in an MHC-haplomatched cohort of MHC-M3+ SIVmac239+ Mauritian cynomolgus macaques (MCMs) which were treated with ART at two weeks postinfection, they observed very high levels of posttreatment control for up to 7 months after the cessation of therapy. This control was not due to a selection of a replication-incompetent virus, because they could document replication competent SIV in these postreatemnt controllers using quantitative viral outgrowth assays. They also report that in vivo depletion of CD8α+ cells induced rebound in all animals, and suggest that this could indicate that the observed PTC was mediated, at least in part, by CD8α+ cells. They also performed IPDA to measure the size of the intact viral reservoir and report that in these macaques there are significantly smaller viral reservoirs two wpi than a cohort of identically infected rhesus macaques, a population that rarely develops PTC. They conclude that an unusually small viral reservoir is a hallmark among SIV+ MCMs. They also established that PTC was associated with a reduced frequency of CD4+ and CD8+ lymphocyte subsets expressing exhaustion markers. Together, these results suggest a combination of small reservoirs and immune-mediated virus suppression contribute to PTC in MCMs.

The manuscript is concise, well written that results are of potentially high impact and, altogether my recommendation is of acceptance after several minor modifications.

We thank Dr. Apetrei for his thoughtful review of this manuscript and encouraging comments. We have addressed the suggestions and recommendations made in the following ways (in blue):

Major Comments: None

Minor Comments:

1. The CD8+ cell depletion experiments induce a massive proliferation of the CD4+ T cells that can be monitored by the measurement of the CD4+ T cell fraction expressing the proliferation marker Ki—67. This increased CD4 proliferation could contribute at least in part to virus reactivation upon depletion. In my opinion, it is important that we monitor the dynamics of the KI-67 expression by the T cells after the CD8 cell depletion.

We agree that proliferation of CD4+ T cells after CD8 depletion could contribute to the observed rebound viremia. If CD4+ T cells harboring reservoir virus proliferated after CD8α depletion, this could have contributed to virus production in addition to the removal of CD8-mediated virus suppression. Interestingly, Sutton et al ([PMC6639280](http://www.ncbi.nlm.nih.gov/pmc/articles/pmc6639280/)) found that CD8β depletion in MCMs infected with a live attenuated SIV did not dramatically increase the frequency of proliferating (ki-67+) CD4+ T cells (naive, TCM, or TEM) compared to animals that received a control IgG1 antibody. This could be a consequence of using the CD8β-depleting antibody rather than the CD8α-depleting antibody we used in the present study. However, these results suggest that while CD8 depletion can cause CD4+ T cell proliferation as Dr. Apetrei rightly points out, not all CD8 depletions cause massive CD4+ T cell expansion.

We have included a figure below for the reviewers that we do not feel comfortable showing in the manuscript. We stained pre-and post-depletion samples in two independent experiments, one shown in Figure 1 E-G and the other shown below. Unfortunately, we mistakenly included an anti-CD8 antibody in the panel shown to the reviewer below that interacted with the anti-CD8 depleting antibody, leading to epitope masking. We thus did not include that data in the manuscript and repeated the experiment with a different anti-CD8 antibody (clone DK25) to avoid epitope masking in the figures shown in the manuscript. However, the samples stained for Figure 1 in the manuscript did not have CBCs performed, so we were unable to calculate absolute cell counts for the depletion figures in the manuscript

We feel it is important to address the comments raised by the reviewers, so we are presenting this data for the reviewer, but will include it in the manuscript. The plots below are generated from the experiment that used the mistaken anti-CD8 antibody, even though we do not expect the CD4-staining was affected. The plots show that the frequency of CD4+ T cells as a proportion of the CD3+ population increased after CD8 depletion, but the absolute CD4+ T cell counts did not significantly change after CD8 depletion compared to pre-depletion. Of course, it is still possible that the CD4+ T cells did proliferate. Unfortunately, Ki-67 was not included in the panel we used to stain samples in which we monitored depletion of CD8+ T cells and NK cells (Figure 1E, F), and samples taken after CD8α depletion have already been used for other studies for many of the animals, so we cannot assess CD4+ T cell proliferation for the animals described here. However, we agree this is an important point that we will plan to measure in future studies of CD8 depletion in MCMs.

2. One of the reasons for a lower viral reservoir in the MCMs may be that the virus is less well adapted in the MCMs than in the rhesus macaques. In the past there were several instances in which, after a relatively successful acute replication, cross-species transmitted viruses were controlled by the macaque host [i.e., SIVrcm by cynomolgous macaques (Marx), SIVagm by the rhesus macaques (Pandrea) etc]. It might be great to see the dynamics of interferon production during the acute infection in these monkeys, although it is possible that samples might be limited. These issues should at least be discussed.

This is a very good point, and one we did not discuss sufficiently in the previous version. While MCMs, particularly M3+ MCMs, do develop high peak and setpoint SIVmac239 viral loads similar to those observed in RMs, SIVmac251 does appear to be less pathogenic in MCMs than RMs. If SIVmac239M is less well adapted to MCMs than it is to RMs, then this could impact acute reservoir formation. It could also impact the species-specific differences in acute viremia, which we have added additional plots to show in Figure 2 in response to an additional request by Reviewer 4; RMs exhibit more sustained peak viremia whereas MCMs appear to undergo a post-peak decline in viral load more rapidly. We have added a discussion of this topic in lines 343-351.

We agree it would also be interesting to evaluate interferon production during acute infection and the potential impact on reservoir formation dynamics and the differences between rhesus macaques and MCMs. Unfortunately, those samples are unavailable from animals in the present study, but we plan to collect additional samples in future animal studies to assess this possibility. A discussion of this point has been added to lines 333-337.

# Responses to Reviewer #4:

This study by Harwood et al. reports a high frequency of post-treatment controllers in a cohort of SIV-infected MCM expressing at least one copy of the M3 MHC haplotype, treated with ART for ~8 months starting 2 weeks post-infection (wpi). Seven out of eight MCM controlled viremia for extended period of times after ATI despite the demonstrated presence of replication-competent viruses in the reservoir. Following rechallenge with an isogenic virus, some of these PTC were shown to maintain undetectable viremia while CD8+ cell depletion inevitably led to a rebound in viremia. Additional viral loads data are presented from other cohorts of RMs and MCM initiated on ART during chronic infection. Animals from both cohorts of MCM were then regrouped based on ATI outcome to interrogate the parameters associated with PTC.

The manuscript contains a lot of interesting data supporting the specificity of SIV immunopathogenesis in Mauritanian Cynomolgus macaques previously suggested by others. However, data interpretation is at time confusing and complicated by the design of some analyses and by their representation. Most notably, the validity of the comparative analyses is questionable due to important confounding factors including genetic background, route of infection and infectious dose. Considering these limitations, the authors should be more measured in their interpretation of the data and conclusions.

We thank this reviewer for their rigorous and review of this manuscript and suggestions of additional relevant information to include and further clarify. We have addressed the comments raised by this reviewer in the following ways (in blue):

Major Comments:

1. While it is not directly shown, it seems that viremia might have peaked earlier in the MCM than in the RMs. The lower levels of total and intact DNA in MCMs at 2wpi could thus reflect their lower plasma viral load - which maybe be controlled faster due to intrinsic differences in T cells responses. It would be interesting to compare total and intact DNA levels between MCM and RM at the time of peak viremia rather than at 2wpi. Also, showing the longitudinal PVL in the group of RMs (at least 1 and 2wpi) would be informative. The comparison between groups is limited as the RMs did not receive ART.

We appreciate this keen observation. Indeed, the acute viral load dynamics of RMs and MCMs are slightly different. We have observed that RMs exhibit more sustained peak viremia whereas MCMs appear to undergo a post-peak decline in viral load more rapidly. As the reviewer points out, this could be due to differences in acute T cell responses. We agree it would be very interesting to measure the total and intact vDNA levels at peak viremia to determine whether peak viremia coincides with peak vDNA by IPDA. Unfortunately, it would be necessary to collect blood daily between days ~7 and 21 to determine the precise time of peak viremia, which could differ from animal to animal in each cohort, and we do not have all of those samples for the animals described here. We chose 2wpi because this was the last timepoint before the MCMs received ART and we had matched RM sample for this day.

We agree that showing the acute viral loads for both cohorts is beneficial to illustrate the differences in acute viral dynamics between the RMs and MCMs described here, so we have added the acute viral loads to Figure 2 as suggested here and by Reviewer 3. We have also included a plot of the acute viral load area under the curve, which compiles viral loads across the timeframe included, in Figure 2 to strengthen the comparison of these cohorts given the two week limitations recognized by this reviewer. Finally, we have added some data to Figure 2 to illustrate some of the intrinsic differences between MCM and RM CD4+ T cell phenotypes, including memory phenotype and CCR5 expression, that may impact reservoir formation. We have added a discussion of this topic in lines 183-193, 329-330, and 343-351.

2. The cohort of MCM initiated on ART at 8 wpi presents several differences with the group of MCM initiated on ART at 2 wpi that limit its value as a comparative group. Considering the differences in term of route of infection, possibly infectious dose, and the fact that pre-ART levels of total and intact DNA were measured at 2 different time points (2wpi during peak viremia for cohort 1 and at 8wpi for cohort 2, it seems difficult to conclude that “A longer duration of SIV infection prior to ART does not increase the frequency of cells with intact proviral DNA but increases the likelihood of viral rebound” (title of the 5th result paragraph). The same comment applies to the next paragraph title “despite similar reservoir size” and to several other sentences throughout the manuscript claiming a smaller reservoir in cohort 1.

We thank the reviewer for this critique. We recognize that there are multiple differences between the MCM_2wk cohort and the MCM_8wk cohort, including dose and route of SIV challenge and timing of ART initiation, that the reviewer rightly points out. While it would be ideal to only include animals with matched infection and sampling histories, this is not always possible, particularly as these animals become more expensive and difficult to obtain. These animals are also a valuable resource that we aim to use to the fullest extent to maximize what we can learn from the fewest animals. We therefore used all animals knowing they are an imperfect match. We previously highlighted these limitations in the original version (now lines 198-208, 438-444), and we did not want to cause confusion by further emphasizing this point. However, we appreciate the critique and do not want to overstate the results presented. We changed the Result titles mentioned by the reviewer to be more measured in interpretation.

Minor Comments:

1. Regarding the role of the CD8+ cells in the post-treatment control of viremia, it would be informative to include the longitudinal levels of CD8+ T cells and NK cells represented in parallel with the viremia for each MCM individually to show if control is restored when CD8+ T cells return.

We agree it is very interesting to understand the relationship between CD8+ T cell and NK cell return after depletion and the viral load dynamics. We have included plots for seven of the eight animals below depicting longitudinal CD8+ T cells (top plots) or NK cells (bottom plots) and viral load after CD8α depletion below. As expected, post-depletion viral load decline frequently coincides with the return of CD8+ T cells and NK cells. However, our lab is currently preparing a separate manuscript specifically describing the viral dynamics throughout this study, including SIVmac239M barcode analyses and the relationship between lymphocyte return and viral load kinetics depicted below. For this reason, we excluded that data in the present manuscript because this data is essential in discussing the viral dynamics, and we aim to prevent repeating data shown in multiple manuscripts, where possible.

2. It would be best to keep the individual symbol representation of each MCM from cohort 1 and cohort 2 throughout all figures.

We agree that including the animal symbols throughout all figures helps with consistency and clarity in animal designations. We have included each animal symbol in each figure and supplemental figure, adjusting the designations that previously included shape to avoid confusion, except Supplemental Figures 2 and 4 because in Supplemental Figure 2, shape is a necessary identifier to denote temporary groupings, and in Supplemental Figure 4, many of the shapes were indistinguishable, so we left the graphs with bars and dots for clarity.

3. Figure 3A: the schematic indicates that cohort 2 was initiated on ART at 2wpi instead of 8wpi

We thank the reviewer for pointing out that Figure 3A was unclear. We had shown the timeline in months while the viral loads shown in Figure 3B indicated time in weeks. We have converted the timeline depicted in Figure 3A to show time in weeks to maintain consistency throughout this figure.

4. In the discussion, the authors state that “the frequency of bulk and SIV-specific T cells subsets expressing ICIs declined after ART withdrawal in PTC”. This should be rephrased to accurately describe the results.

We have adjusted the sentence highlighted by this reviewer in lines 279-281 to reflect that only some of the bulk, memory, and SIV-specific T cell subsets exhibited a decline in the frequency cells expressing certain ICIs (but not all subsets or ICIs) after ART withdrawal compared to before.

5. In the discussion, the authors state that Bender et al reported levels of intact DNA 10 times higher in RMs than in humans. This article reports levels 10 times higher after 36 weeks of ART in RMs infected with SIVmac251 but similar levels of intact DNA between HIV-infected individuals on long term ART and RM infected with SIVmac239 on ART.

We appreciate the reviewer recognized the comparison of our data to the Bender paper. We thought that the Bender paper did say that SIVmac251-infected rhesus macaques on long term ART had similar numbers of intact viral genomes to SIVmac239-infected rhesus macaques on ART, which were both approximately 10-fold higher than the number detected in HIV-1+ humans on long term ART (Bender 2019 Figure 7a), but perhaps we misunderstood or were not clear in our text. Even so, we recognize that it is difficult to compare levels of viral DNA between species, and also between animals (and humans) infected with different challenge viruses. Therefore, we have added this discussion point and reduced the emphasis on this point to soften the conclusions drawn in lines 359-366.
